# Allele-specific methylation in the *FADS* genomic region in DNA from human saliva, CD4+ cells, and total leukocytes

**DOI:** 10.1186/s13148-018-0480-5

**Published:** 2018-04-06

**Authors:** Elaheh Rahbar, Charlotte Mae K. Waits, Edward H. Kirby, Leslie R. Miller, Hannah C. Ainsworth, Tao Cui, Susan Sergeant, Timothy D. Howard, Carl D. Langefeld, Floyd H. Chilton

**Affiliations:** 1 Department of Biomedical Engineering, Wake Forest University School of Medicine, Virginia-Tech Wake Forest School of Biomedical Engineering and Sciences, 575 N. Patterson Ave. Suite 120, Winston-Salem, NC 27101 USA; 2 Virginia-Tech Wake Forest School of Biomedical Engineering and Sciences, Blacksburg, VA USA; 3 Department of Physiology and Pharmacology, Wake Forest School of Medicine, 575 N. Patterson Ave. Suite 310, Winston-Salem, NC 27101 USA; 4 Department of Biostatistical Sciences, Division of Public Health Sciences, Wake Forest School of Medicine, Medical Center Blvd/525 Vine Street, Winston-Salem, NC 27157-1063 USA; 5 Department of Urology, Wake Forest School of Medicine, 1 Medical Center Blvd, Winston-Salem, NC 27157 USA; 6 Department of Biochemistry, Wake Forest School of Medicine, 1 Medical Center Blvd, Winston-Salem, NC 27157 USA

**Keywords:** CD4+, Saliva, Leukocytes, Whole blood, Omega-6, PUFA, DNA methylation, rs174537, *FADS*

## Abstract

**Background:**

Genetic variants within the fatty acid desaturase (*FADS*) gene cluster (human Chr11) are important regulators of long-chain (LC) polyunsaturated fatty acid (PUFA) biosynthesis in the liver and consequently have been associated with circulating LC-PUFA levels. More recently, epigenetic modifications such as DNA methylation, particularly within the *FADS* cluster, have been shown to affect LC-PUFA levels. Our lab previously demonstrated strong associations of allele-specific methylation (ASM) between a single nucleotide polymorphism (SNP) rs174537 and CpG sites across the *FADS* region in human liver tissues. Given that epigenetic signatures are tissue-specific, we aimed to evaluate the methylation status and ASM associations between rs174537 and DNA methylation obtained from human saliva, CD4+ cells and total leukocytes derived from whole blood. The goals were to (1) determine if DNA methylation from these peripheral samples would display similar ASM trends as previously observed in human liver tissues and (2) evaluate the associations between DNA methylation and circulating LC-PUFAs.

**Results:**

DNA methylation at six CpG sites spanning *FADS1* and *FADS2* promoter regions and a putative *FADS* enhancer region were determined in two Caucasian cohorts of healthy volunteers: leukocytes in cohort 1 (*n* = 89, median age = 43, 35% male) and saliva and CD4+ cells in cohort 2 (*n* = 32, median age = 41, 41% male). Significant ASM between rs174537 and DNA methylation at three CpG sites located in the *FADS2* promoter region (i.e., chr11:61594865, chr11:61594876, chr11:61594907) and one CpG site in the putative enhancer region (chr11:61587979) were observed with leukocytes. In CD4+ cells, significant ASM was observed at CpG sites chr11:61594876 and chr11:61584894. Genotype at rs174537 was significantly associated with DNA methylation from leukocytes. Similar trends were observed with CD4+ cells, but not with saliva. DNA methylation from leukocytes and CD4+ cells also significantly correlated with circulating omega-6 LC-PUFAs.

**Conclusions:**

We observed significant ASM between rs174537 and DNA methylation at key regulatory regions in the *FADS* region from leukocyte and CD4+ cells. DNA methylation from leukocytes also correlated with circulating omega-6 LC-PUFAs. These results support the use of peripheral whole blood samples, with leukocytes showing the most promise for future nutrigenomic studies evaluating epigenetic modifications affecting LC-PUFA biosynthesis in humans.

**Electronic supplementary material:**

The online version of this article (10.1186/s13148-018-0480-5) contains supplementary material, which is available to authorized users.

## Background

Polyunsaturated fatty acids (PUFAs) play important roles in a wide variety of physiologic and homeostatic processes ranging from the biophysical properties responsible for cell membrane function and energy production to an array of signaling events mediated by bioactive lipid metabolites [1, 2]. Unsurprisingly, PUFAs play a critical role in a wide array of human diseases, including cardiovascular disease [3–5], metabolic syndromes [6], and cancer [7, 8]. Several genetic variants near and within the fatty acid desaturase (*FADS*) gene cluster (chr11: 61,540,615-61,664,170) are strongly associated with the capacity to convert 18 carbon dietary PUFAs to biologically active long-chain (LC) PUFAs (i.e., ≥ 20 carbons). Notably, a single nucleotide polymorphism (SNP) rs174537, residing ~ 15 kb downstream of *FADS1*, displays a strong association (*p* < 10^−40^) with the omega-6 (also referred to as n-6) LC-PUFA, arachidonic acid (ARA; C20:4n-6), and product to precursor ratios (e.g., ARA/DGLA, DGLA: dihomo-γ-linolenic acid; C20:3n-6) measured in both whole blood and tissue specimens [6, 9, 10]. This is particularly important because ARA is a precursor to numerous bioactive lipid metabolites including eicosanoids and endocannabinoids which can contribute to the development and progression of immune responses as well as acute and chronic inflammatory diseases. In addition to LC-PUFA levels themselves, genetic variation within the *FADS* cluster is associated with numerous human phenotypes, including inflammatory [11] and cardiovascular disorders [12, 13], insulin resistance [14], perinatal depression [15], atopic diseases [16–18], attention deficit disorder/hyperactivity, intelligence and memory in children [19, 20].

The molecular mechanisms by which *FADS* genetic variants affect LC-PUFA biosynthesis and overall LC-PUFA levels remain unclear. Expression quantitative trait loci (eQTL) mapping have shown that some *FADS* variation is associated with *FADS* gene expression levels [10, 21]. However, changes in *FADS* gene expression levels could occur in several ways, such as altering the regulatory landscape (e.g., promoter or enhancer) of a gene, alternative RNA splicing, transcript degradation, or transcription of non-coding RNA. Additionally, epigenetic modifications such as DNA methylation or histone modifications can influence gene expression levels [22]. DNA methylation is known to alter gene expression, especially when located in critically regulatory regions, such as a promoter or enhancer [22, 23]. We recently performed a genome-wide, allele-specific (GWAS) methylation (ASM) analysis with rs174537 to test the hypothesis that rs174537 is associated with DNA methylation levels in human liver tissues, since the liver is believed to be the primary organ for fatty acid biosynthesis [10, 24]. Indeed, we observed significant associations between rs174537 and methylation at eight CpG sites, spanning the *FADS1* promoter, a putative enhancer, and *FADS2* promoter region, all located in a 12-kb regulatory region between *FADS1* and *FADS2* genes [24]. However, since DNA methylation is known to be highly tissue-dependent, the objective of this study was to determine if the strong ASM between rs174537 and these key CpG sites could be detected in easily accessible peripheral samples and biological fluids, such as saliva and whole blood. A secondary goal was to determine the associations between DNA methylation from these peripheral biological specimens and circulating PUFA levels.

## Methods

### Subject recruitment and sample collection

Healthy adult subjects (21–65 years old) were recruited by two mechanisms referred to as cohort 1 and cohort 2. Both studies were approved by the Wake Forest School of Medicine Institutional Review Board (IRB#s 00016822 and 00006156, respectively), and all participants provided written and informed consent for their respective study, as well as for future research use of their archived biospecimens. Exclusion criteria included diagnosis of major disease, such as diabetes, cardiovascular, or inflammatory disease. Additionally, participants were excluded if they used tobacco products and fish or botanical seed oil supplements or if they had received a tattoo or body piercing within 1 year of enrollment. Individuals with elevated fasting triglycerides (> 150 mg/dl), fasting blood glucose (> 125 mg/dl), and resting blood pressure (> 130/90) were also excluded. Participants had to be > 50 kg and have BMIs between 19 and 30. Individuals who took nonsteroidal anti-inflammatory drugs (NSAIDs) or had a common cold required 1- and 2-week postponement, respectively.

In cohort 1, 89 subjects provided fasting whole blood samples collected in purple-top Vacutainer® tubes containing EDTA (Cat# 367861, Becton Dickinson, Franklin Lakes, NJ) and in a separate marbled red and gray Vacutainer® tube without an anti-coagulant (Cat# 367988). Total leukocytes in the EDTA blood tube were the source of DNA, which was extracted using standard molecular biology techniques as described in the Gentra Puregene Handbook, third edition [25]. Serum was obtained by centrifugation of the second blood tube (i.e., no anti-coagulant) and stored at − 20 °C for future fatty acid quantification.

In cohort 2, 32 subjects provided matched whole blood and saliva samples. Whole blood (180 ml) was collected in sterile, heparinized syringes. A small volume (5 ml) of blood was centrifuged (10 min at 1000 rpm) to isolate plasma, which was subsequently frozen for future quantification of circulating fatty acids. The remainder of the blood was processed to isolate peripheral blood mononuclear cells (PBMCs) [26]. Briefly, a leukocyte-enriched suspension was obtained after mixing whole blood with Isolymph (Gallard-Schlesinger Industries, Carle Place, NY). PBMCs were recovered from fractionation over Isolymph after centrifugation. CD4+ cells were further isolated from PBMCs using positive selection with a MACS Miltenyi Biotec (San Diego, CA) CD4 MicroBeads (human) kit according to the manufacturer’s protocol. DNA was extracted from these CD4+ cells using standard molecular biology techniques [25]. Matched saliva samples were collected in cohort 2; after a water rinse of the mouth, subjects provided a saliva (~ 2 ml) using a DNA Genotek Oragene (OGR-500, Ottawa, ON, Canada) collection device. Saliva-derived DNA was extracted following the manufacturer’s standard protocol.

### Fatty acid quantification

Total circulating (cohort 1: serum, cohort 2: plasma) fatty acids were assessed as fatty acid methyl esters (FAME) analyzed by gas chromatography with flame ionization detection (GC-FID) using a Hewlett Packard 5890 instrument system with an Agilent J&W DB-23 column (30 m, 0.25 mm ID, 0.25 μm film) fitted with an inert pre-column (1 m, 0.53 mm ID) for cool on-column injection as previously described [27]. Independent experiments from our lab and others have shown that the fatty acid profiles of serum and plasma from anti-coagulated blood (heparin or EDTA) are comparable [28, 29]. Fatty acids were cleaved from complex lipids and converted to methyl esters in duplicate samples (100 μl) utilizing a modification of the protocol developed by Metcalfe et al. [30] and Sergeant et al. [27]. Fatty acids in samples were identified based on retention times of commercially available standards. Triheptadecanoin (100 μg; NuChek Prep, Elysian, MN) was used as an internal standard. Fatty acid peaks (23–29 peaks) were identified and accounted for > 99% of the total fatty acids in the sample. Fatty acid data are presented as the mass percent of total fatty acids from serum (cohort 1) or plasma (cohort 2).

### Genotyping SNP rs174537

Following DNA isolation and quantification, rs174537 genotyping was performed using the Sequenom iPLEX® genotyping system using the manufacturer’s instructions (Sequenom, Inc., San Diego, CA) as previously described [10]. Total leukocyte-derived DNA (for cohort 1) and DNA derived from CD4+ cells (for cohort 2) were used for genotyping.

### DNA methylation quantification

The methylation status of DNA obtained from total leukocytes (cohort 1) and CD4+ cells and saliva (cohort 2) was assessed using pyrosequencing, as previously described [31]. Based on our previous studies, DNA methylation was quantified at six key CpG sites between *FADS1* and *FADS2.* These sites were previously identified to have strong ASM with rs174537 from liver tissues [10, 24]. While our previous paper identified eight CpG sites, we were limited by sample material to analyze six out of the eight CpG sites in this paper. Specifically, we quantitated two CpG sites in the *FADS1* promoter region (chr11:61584836 and chr11:61584894), one CpG site located within the putative enhancer region between *FADS1* and *FADS2* (chr11:61587979, which is cg27386326), and three CpG sites in the *FADS2* promoter region (chr11:61594865, chr11:61594876, and chr11:61594907).

Briefly, genomic DNA obtained from each peripheral sample (1 μg; total leukocytes, CD4+-derived cells, and saliva) were treated with sodium bisulfite using the EZ 96-DNA methylation kit (Zymo Research, Irvine, CA) following the manufacturer’s standard protocol. To assay the cg27386326 CpG site, pyrosequencing with the cg27386326_04_PM assay was used with a PyroMark Q96 MD (Qiagen, Inc.; Germantown, MD). Custom primer sets were designed for the two CpG sites located in the *FADS1* promoter region and three CpG sites in the *FADS2* promoter region (Qiagen, Inc.). Methylation was then quantitated with Pyro Q-CpG (version 1.0.9; Biotage, Inc.). In total, six CpG sites were successfully quantitated each from the saliva, CD4+, and total leukocyte samples. Additional file 1: Table S1 provides a list of the primers and sequencing probes used to quantitate these sites.

### Statistical analysis

Means and standard deviations of percent DNA methylation measured at each CpG site and for each DNA source (i.e., leukocytes, CD4+, and saliva) were calculated and evaluated for statistical differences using Wilcoxon-Mann-Whitney tests. A Wilcoxon matched-pairs signed-rank test was used to evaluate differences between matched saliva- and CD4+-derived DNA methylation levels in cohort 2. DNA methylation from each CpG site was evaluated for an association with genotype at rs174537 using a linear regression model adjusted for age and gender. Given that both cohorts were limited to Caucasians, no adjustments for race were made. Genotypes for rs174537 were coded for additive and dominant genetic models, relative to the T allele. Due to limited individuals with TT genotype in cohort 2 (i.e. *n* = 2), the genotype comparisons were made between GG (homozygous dominant, referent group) and “GT/TT.” CpG site associations in the two cohorts were then analyzed. Benjamini-Hochberg false discovery rate (BH-FDR) *p* values were calculated and reported along with the raw *p* values. Significance was set at the 0.05 BH-FDR level. In cohort 2, where matched saliva and CD4+ samples were obtained, we computed pairwise correlation analysis (Spearman correlations) to determine the level of association between DNA methylation from the two peripheral fluid types. Similarly, Spearman rank correlations were conducted to evaluate associations between circulating PUFAs and DNA methylation levels for each DNA source. All statistical analyses were conducted using commercially available software (STATA, SAS 9.4) and open source statistical software (R).

## Results

A total of 121 Caucasian subjects were included in the analysis, 89 from cohort 1 and 32 from cohort 2. Both cohorts had similar age and gender distributions, albeit cohort 2 lacked age information on one individual. The median age for cohort 1 was 43 years with an interquartile range (IQR) of 30–53 years. The median age for cohort 2 was 41 years (IQR 27–55). Cohort 1 consisted of 31 males (35%), and cohort 2 included 13 males (41%).

Means and standard deviations of percent DNA methylation measured at each CpG site and for each DNA source (i.e., leukocytes, CD4+, and saliva) are presented in Table 1. Pyrosequencing of CD4+ cell DNA was not successful in some samples due to low sample volume, as indicated by the number missing in Table 1. DNA methylation levels from the CpG sites located in the *FADS1* and *FADS2* promoter regions were generally higher in DNA derived from CD4+ cells compared to that obtained from total blood leukocytes and saliva (Table 1). In fact, DNA methylation levels at CpG sites located in the putative enhancer and *FADS2* promoter regions derived from CD4+ cells were significantly higher than their matched saliva counterparts (denoted by *α* in Table 1). Interestingly, DNA methylation at the CpG site located in the putative enhancer region (i.e., chr11:61587979 (cg27386326)) was highest in DNA extracted from total blood leukocyte samples. DNA methylation levels quantified from total leukocytes at this CpG site was comparable to the levels obtained from liver tissue specimens, as previously published [10, 24]. Consistently, the degree of DNA methylation was significantly different between DNA sources, as seen in Table 1.

**Table 1:** Summary of percent DNA methylation at each CpG site for DNA extracted from total leukocytes (cohort 1) and CD4+ cells and saliva (cohort 2)

CpG site		Cohort 1 (*N* = 89)	Cohort 2 (*N* = 32)		
		Total leukocytes	CD4+		Saliva
				# Missing	
*FADS1* promoter	chr11:61584836	4.93 ± 0.90	7.61 ± 1.53^α^*	*N* = 7	8.59 ± 2.17*
	chr11:61584894	11.48 ± 2.39	39.66 ± 4.33*	*N* = 7	25.84 ± 10.74*
Putative enhancer region	chr11:61587979 (cg27386326)	81.22 ± 7.84	68.71 ± 24.20^α^*	*N* = 2	53.94 ± 15.67*
*FADS2* promoter	chr11:61594865	11.80 ± 4.21	30.90 ± 9.33^α^*	*N* = 6	23.41 ± 8.26*
	chr11:61594876	12.46 ± 4.51	34.82 ± 9.33^α^*	*N* = 6	23.94 ± 9.12*
	chr11:61594907	12.71 ± 4.93	38.60 ± 12.89^α^*	*N* = 2	16.86 ± 7.18*

Means ± standard deviations are reported. The number missing in CD4+ cells are a result of insufficient DNA volume for pyrosequencing. DNA methylation levels measured in cohort 2 (i.e., CD4+ and saliva) were significantly different compared to DNA methylation derived from leukocytes in cohort 1, as indicated by asterisks (*p* < 0.05). Significant differences between matched saliva and CD4+ samples in cohort 2 are denoted by *α*

### Correlations between saliva- and CD4+-derived DNA methylation at CpG sites in FADS2 promoter region

Using paired samples from cohort 2, we observed DNA methylation from saliva-derived DNA significantly correlated to CD4+-derived DNA, particularly at CpG site chr11:61594865 located in the *FADS2* promoter region (rho = 0.435, *p* = 0.026), indicating DNA methylation in CD4+ cells explain approximately 20% of the variation in DNA methylation in saliva. Thus, these data indicate there is a modest shared and significant independent DNA methylation variation in these two sources of DNA within the same individual. Notably, the degree of DNA methylation was consistently lower in DNA derived from saliva samples compared to DNA derived from CD4+ cells for CpG sites evaluated in this study (Table 1).

### Allele-specific methylation detected in DNA derived from leukocytes and CD4+ cells with SNP rs174537 and CpG sites located in the FADS2 promoter region

To assess the association of rs174537 with the methylation status of the six selected CpG sites, a linear regression was computed, adjusting for age and gender for each DNA source. We observed significant ASM between DNA methylation quantitated from the total leukocytes at three measured CpG sites across the *FADS2* promoter and one CpG site in the putative enhancer region (Table 2). In particular, three CpG sites located in the *FADS2* promoter region (i.e., chr11:61594865, chr11:61594876, and chr11:61594907) exhibited the strongest ASM associations with rs174537 based on the FDR *p* value < 0.05 in leukocyte-derived samples (Table 2). DNA methylation levels at CpG site (chr11:61587979, also known as cg27386326) in the putative enhancer region also displayed significant ASM in leukocyte-derived samples (Table 2). In CD4+ cells, we observed significant ASM with rs174537 and CpG sites chr11:61584894 (located in *FADS1* promoter) and chr11:61594876 (located in *FADS2* promoter) (Table 3). No significant ASM associations were observed (FDR > 0.05) for the human saliva specimens.

**Table 2:** Significant ASM associations with rs174537 in leukocyte-derived samples from cohort 1

Location	CpG site	Estimate	Std. Err.	*R* ^2^	Raw *p* value	FDR *p* value
*FADS1* promoter	chr11:61584836	0.001	0.002	0.03	4.83E−01	4.83E−01
	chr11:61584894	− 0.010	0.005	0.07	6.08E−02	7.29E−02
Putative enhancer region	chr11:61587979* (cg27386326)	0.038	0.016	0.07	2.54E−02	3.80E−02
*FADS2* promoter	chr11:61594865*	− 0.029	0.009	0.14	9.29E−04	1.86E−03
	chr11:61594876*	− 0.047	0.008	0.27	3.07E−07	9.20E−07
	chr11:61594907*	− 0.051	0.009	0.27	2.52E−07	9.20E−07

Genetic trend test adjusted for age and gender revealed significant ASM associations (*FDR *p* value < 0.05) with DNA methylation quantitated from whole blood-derived total leukocytes

**Table 3:** Significant ASM associations with rs174537 in CD4+ cell-derived samples from cohort 2

Location	CpG site	Estimate	Std. Err.	*R* ^2^	Raw *p* value	FDR *p* value
*FADS1* promoter	chr11:61584836	− 0.009	0.007	0.19	1.91E−01	3.18E−01
	chr11:61584894*	− 0.050	0.016	0.37	6.78E−03	3.70E−02
Putative enhancer region	chr11:61587979 (cg27386326)	0.052	0.087	0.06	5.57E−01	6.40E−01
*FADS2* promoter	chr11:61594865	− 0.075	0.037	0.33	5.33E−02	1.78E−01
	chr11:61594876*	− 0.101	0.034	0.43	7.46E−03	3.70E−02
	chr11:61594907	− 0.069	0.044	0.35	1.31E−01	2.88E−01

Genetic trend test adjusted for age and gender revealed significant ASM associations (*FDR *p* value < 0.05) with DNA methylation quantitated from whole blood-derived CD4+ cells

**Fig. 1:**
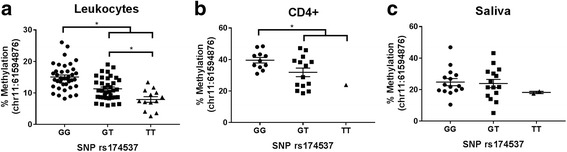
Allele-specific methylation with rs174537 and CpG site (chr11:61594876) located in the *FADS2* promoter region. Mean ± SEM are displayed, along with the spread of raw data. Percent DNA methylation from **a** leukocytes (cohort 1), **b** CD4+ cells (cohort 2), and **c** saliva (cohort 2). Asterisks represent statistically significant genotypic differences (*p* < 0.05) using a linear regression model adjusted for age and gender. For cohort 2, GT/TT were combined due to limited sample size

Closer examination of DNA methylation derived from total blood leukocytes (Fig. 1a) demonstrated significant genotypic effects and marked differences in DNA methylation levels across genotype consistent with an additive genetic model at all three CpG sites within the *FADS2* promoter region. Individuals with the GG genotype at rs174537 had the highest mean level (± standard error) of DNA methylation at CpG site chr11:61594876 (15.06 ± 0.66%), followed by GT (11.31 ± 0.58%) and TT (7.93 ± 0.89%), illustrated in Fig. 1a. Similarly, for CD4+ cells (Fig. 1b), we observed significantly higher DNA methylation levels in individuals with GG genotype at rs174537 (39.58 ± 1.74%) compared to GT/TT individuals (31.34 ± 2.59%). No significant genotypic effects on percent DNA methylation derived from saliva (Fig. 1c) were observed.

**Fig. 2:**
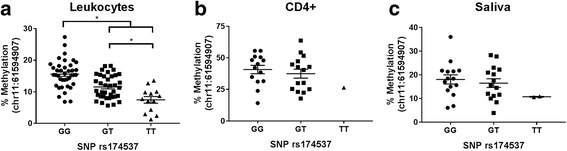
Allele-specific methylation with rs174537 and CpG site (chr11:61594907) located in the *FADS2* promoter region. Percent DNA methylation from **a** leukocytes (cohort 1), **b** CD4+ cells (cohort 2), and **c** saliva (cohort 2). Asterisks represent statistically significant differences (*p* < 0.05) using a linear regression model adjusted for age and gender. For cohort 2, GT/TT were combined due to limited sample size. Mean ± SEM are displayed

For the CpG site chr11:61594907, DNA derived from total blood leukocytes again demonstrated the strongest effect of genotype on methylation (Fig. 2a). Like before, individuals homozygous with the G allele (i.e., GG at rs174537) exhibited the highest levels of DNA methylation compared to those carrying the T allele (i.e., GT and TT, *p* < 0.05). This is not surprising, since all three CpG sites (i.e., chr11:61594865, chr11:61594876, and chr11:61594907) located in the *FADS2* promoter region displayed significant ASM in the leukocyte samples. However, we observed no significant genotypic effects on percent DNA methylation derived from CD4+ cells (Fig. 2b) or saliva (Fig. 2c) at this site, though there appears to be a trend for the additive model in this small cohort. We attribute the weaker statistical significance in ASM relationships from cohort 2 (i.e., saliva and CD4+ cells) at this site to the limited and smaller sample size (*N* = 32), with only two TT subjects. It should also be noted that there was some degree of collinearity between DNA methylation levels at these three CpG sites (i.e., chr11:61594865, chr11:61594876, and chr11:61594907). Interestingly, significant ASM was observed in both leukocytes and CD4+ cells at CpG site chr11:61594876 located in the *FADS2* promoter region.

**Fig. 3:**
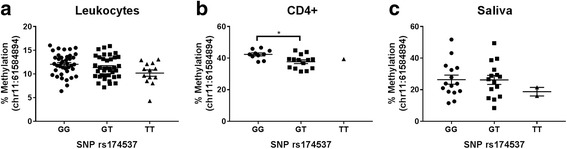
Allele-specific methylation with rs174537 and CpG site (chr11:61584894) located in the *FADS1* promoter region. Percent DNA methylation from **a** leukocytes (cohort 1), **b** CD4+ cells (cohort 2), and **c** saliva (cohort 2). Asterisks represent statistically significant differences (*p* < 0.05) using a linear regression model adjusted for age and gender. For cohort 2, GT/TT were combined due to limited sample size. Mean ± SEM are displayed

Lastly, CD4+ cells displayed significant ASM between rs174537 and a CpG site in the *FADS1* promoter region (Chr11:61584894), with GG genotype being associated with the highest level of DNA methylation (Fig. 3). Despite showing similar trends, this behavior was not statistically significant in leukocyte- or saliva-based samples.

These results are in agreement with our previous studies that demonstrated DNA methylation at CpG sites in the *FADS2* promoter region was significantly associated with genotype at rs174537 in human liver tissues [10, 24]. In addition, genotype at rs174537 is associated with the level of circulating n-6 LC-PUFAs specifically ARA and ARA/DGLA ratio, in both plasma and serum, which is consistent with previous published studies [9, 31, 32] (Additional file 2: Figure S1).

### DNA methylation from leukocytes and CD4+ cells correlate with circulating n-6 LC-PUFAs and ARA/DGLA ratio

Total serum (cohort 1) and plasma (cohort 2) fatty acid levels were determined and are summarized as percent totals in Additional file 3: Table S2. Given that these were healthy volunteers, no significant difference in circulating fatty acids were observed between study cohorts. In fact, these fatty acid levels were similar to those reported by Lee et al., who measured serum fatty acid levels in 310 Caucasian men [33].

**Fig. 4:**
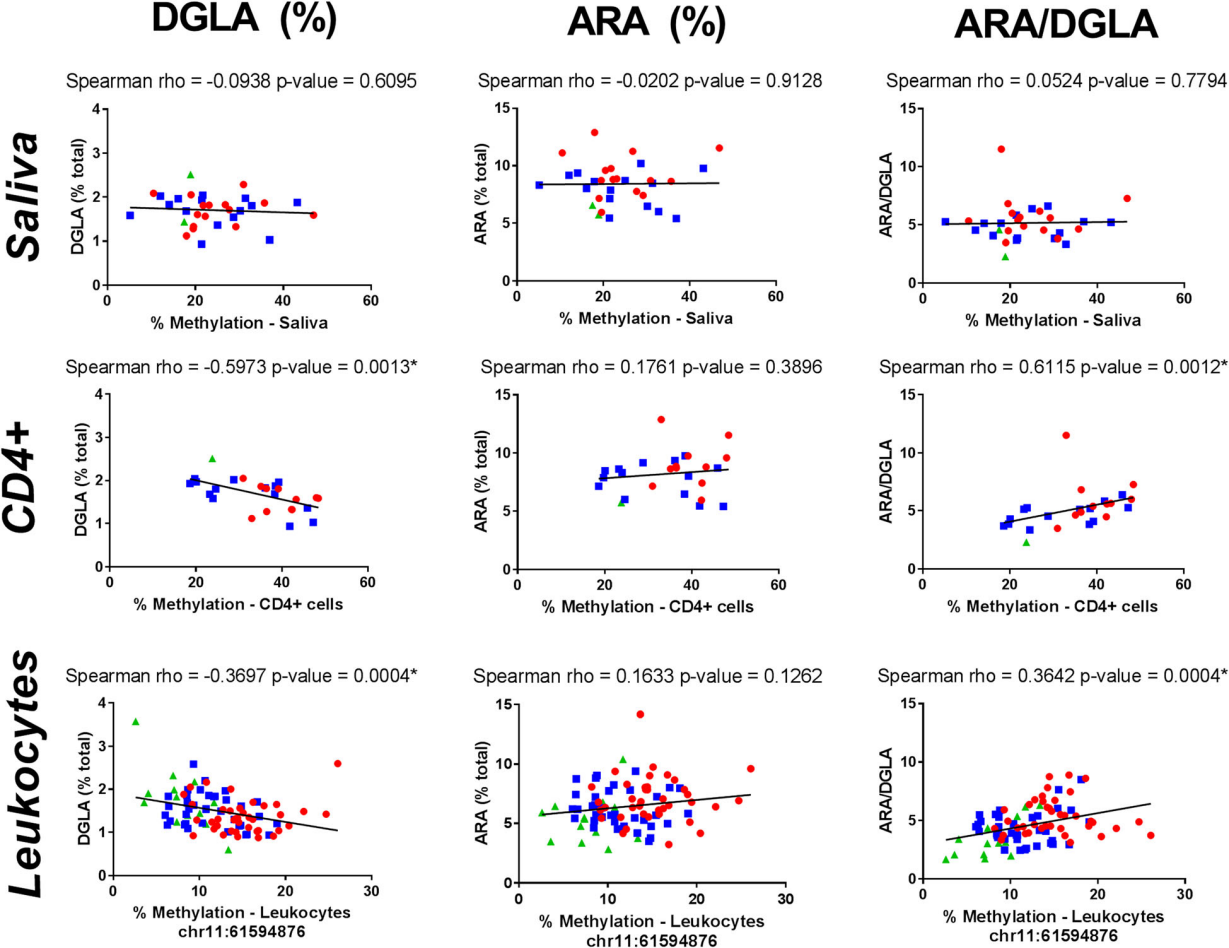
Significant associations between DNA methylation at CpG site chr11:61594876 located in the *FADS2* promoter region and circulating n-6 LC-PUFAs. Spearman correlations with rho and *p* values reported. Individuals with GG genotype at rs174537 are illustrated by red circles, GT blue squares, and TT green triangles

In general, we observed that CD4+ cell-derived and leukocyte-derived DNA methylation levels in the *FADS2* promoter region were good indicators of circulating n-6 LC-PUFA levels. Specifically, DNA methylation all three CpG sites in the *FADS2* promoter (chr11:61594865, chr11:61594876, and chr11:61594907) were inversely associated with circulating DGLA levels (Fig. 4). The strongest correlations were between DNA methylation from CD4+ cells at CpG site (chr11:61594876) located in the *FADS2* promoter region and circulating DGLA levels (Fig. 4). Higher DNA methylation levels were associated with lower circulating DGLA levels (rho = − 0.60, *p* = 0.0013, Fig. 4). This relationship between DNA methylation from leukocytes (cohort 1) and CD4+ cells (cohort 2) and circulating DGLA levels was consistent in all three CpG sites in the *FADS2* promoter (i.e., chr11:61594865, chr11:61594876, and chr11:61594907).

This relationship is flipped when comparing DNA methylation to circulating ARA/DGLA ratio (Fig. 4). Higher DNA methylation levels were associated with higher ARA/DGLA ratios. Again, this behavior was consistent at all three CpG sites in the *FADS2* promoter region (chr11:61594865, chr11:61594876, and chr11:61594907) with DNA derived from total blood leukocytes (cohort 1) and CD4+ cells (cohort 2), as illustrated in Fig. 4.

## Discussion

In this study, we successfully quantitated the methylation status of six key CpG sites located across the *FADS* region from DNA derived from human saliva, CD4+ cells, and total leukocytes. Highly significant ASM between rs174537 and DNA methylation were observed in leukocyte-derived samples at all three CpG sites in the *FADS2* promoter region. These findings are consistent with previous reports of ASM with DNA methylation from human liver tissues [10, 24]. Specifically, DNA methylation from leukocytes demonstrated the strongest genotypic differences compared to CD4+ cells and saliva. Whole blood-derived samples (i.e., leukocytes and CD4+ cells) also exhibited strong associations with levels of the circulating n-6 LC-PUFA, DGLA, and n-6 LC-PUFA product to precursor ratios (e.g., ARA/DGLA). This latter parameter measures the efficiency by which DGLA is converted to ARA through the *FADS1* (delta 5 desaturase) step. Altogether, the data from this study demonstrate the feasibility of using easily accessible whole blood-derived samples to quantitate DNA methylation and probe for epigenetic modifications and ASM associations impacting LC-PUFA biosynthesis.

This study builds upon previous data from our lab and emerging literature by others supporting the notion that SNPs, such as rs174537, and other SNPs in high linkage disequilibrium (LD), and dietary PUFAs can impact the epigenetic regulation of LC-PUFA biosynthesis in humans [3, 5, 10, 21, 24, 31, 32, 34–38]. The CpG sites located in the *FADS2* promoter region not only demonstrated significant ASM, but also displayed strong associations with circulating plasma and serum n-6 LC-PUFAs. Interestingly, the methylation status of these CpG sites and others located in the *FADS2* promoter region have been shown to be affected by dietary changes. Specifically, Hoile et al. observed significant changes in DNA methylation at chr11:61594876 and chr11:61594907 (*FADS2* promoter region; identified and noted as transcription start site (TSS) -806 and -775, respectively, in Hoile et al.’s paper) in response to the modest omega-3 (n-3) fatty acid supplementation in the form of n-3 LC-PUFAs (i.e., eicosapentaenoic acid (EPA, 20:5n-3); docosapentaenoic acid (DPA, 22:5n-3); docosahexaenoic acid (DHA, 22:6n-3)) or olive oil [34]. In that study, n-3 LC-PUFA supplementation induced an increase in methylation at chr11:61594876 (i.e., TSS -806 in Hoile et al.’s paper) in males, but no significant change in females. Contrastingly, DNA methylation at chr11:61594907 (*FADS2* promoter region) increased in females post n-3 LC-PUFA supplementation, whereas males experienced a decrease in methylation at this site [34]. Most importantly, they observed significant associations between the DNA methylation status and mRNA transcript levels, irrespective of gender or dietary supplement. Notably, methylation status at CpG sites chr11:61594876 and chr11:61594907 (TSS -806 and -775 from Hoile et al.’s paper) were negatively associated with the level of *FADS2* transcript, suggesting some level of epigenetic regulation of these genes [34].

In a similar rodent study, increasing maternal fat intake was associated with increasing methylation levels within the *Fads2* region (up to 20%) in hepatic tissue [39]. Further, *Fads2* mRNA expression levels correlated negatively with methylation status, such that higher maternal fat intake was associated with lower *Fads2* mRNA expression levels in the rodent offspring, regardless of sex. Thus, epigenetic regulation of *Fads2* could contribute to both short- and long-term PUFA biosynthesis. In the current study, we found chr11:61594876 to show great promise as a candidate for further investigation as it demonstrated significant ASM with rs174537 in both leukocyte and CD4+ samples, while adjusting for age and gender. DNA methylation at this site also demonstrated significant associations with circulating n-6 LC-PUFAs levels. Importantly, there was some collinearity in the level of methylation between CpG sites chr11:61594876 and chr11:61594907; thus, further investigation is needed to determine the mechanism by which these CpG sites sense dietary changes and influence *FADS2* gene expression. Finally, outside of the *FADS2* promoter region, we observed significant ASM only in CD4+ cells at chr11:61584894, located in the *FADS1* promoter region. While this behavior was similar in leukocytes, it was not replicated with statistical significance.

In general, DNA methylation derived from blood leukocytes in cohort 1 were significantly associated with circulating n-6 LC-PUFAs; the strongest correlations were observed with DGLA and the ARA/DGLA ratio. No statistically significant associations between DNA methylation and circulating n-3 LC-PUFAs were observed in our study cohorts, potentially due to hypomethylation of CpG sites located in the *FADS1* and *FADS2* promoter regions and relatively low levels of circulating n-3 LC-PUFAs due to the fasting nature of subjects in our study cohorts. While we did not observe associations with n-3 LC-PUFAs, other investigators have demonstrated significant associations between n-3 LC-PUFA EPA and n-6 LC-PUFA ARA levels with global DNA methylation [35, 40]. Tremblay et al. demonstrated significant changes in DNA methylation levels (derived from blood leukocytes) following a n-3 PUFA supplementation diet [36]. In a clinical trial of mild to moderate Alzheimer’s disease patients, Karimi et al. found that increasing levels of EPA, but not DHA, were associated with decreased methylation from DNA obtained from blood leukocytes, regardless of cognition scores or gender [35]. Further, Silva-Martinez et al. reported that ARA exposure results in an increase in global DNA methylation levels in human THP-1 monocytes [37]. Therefore, more work is needed to determine how epigenetic modifications and specifically which CpG sites are most associated with dietary exposure of n-3 vs. n-6 LC-PUFAs. This is particularly important as we try to develop methods to monitor the inherent capacity of individuals to convert dietary 18 carbon PUFAs found in high concentrations in the modern Western diet to biologically active LC-PUFAs based on both genetic variation and dietary exposure. Ideally, such methods would utilize DNA from easily accessible cells or tissues, demonstrated by us and these studies. Additionally, the use of total leukocytes and CD4+ cells for quantitating epigenetic modifications may allow one to further investigate the mechanisms by which dietary PUFA exposure impacts inflammatory processes in various disease states.

While this study confirmed significant ASM with CpG sites in the *FADS2* promoter region, we should not overlook the *FADS1* promoter region and other genes that may regulate PUFA biosynthesis. Hypomethylation of CpG sites in the *FADS1* promoter region, as observed in this study, may play an equally important role in regulating *FADS* gene expression and enzyme activity. We also recognize that other genes and regions involved in LC-PUFA biosynthesis, such as *ELOVL*, may be susceptible to epigenetic modifications influencing PUFA biosynthesis and metabolism. For example, methylation levels at two CpGs located in the distal promoter region of the histone deacetylase 4 (*HDAC4*) gene have been previously shown to be inversely associated with ARA in adult males [37, 40]. Another limitation of this study is that only site-specific DNA methylation was quantitated and both cohorts were limited to Caucasians. Future studies should consider evaluating changes in histone modifications in addition to DNA methylation at a global level, since histone modifications are more dynamically responsive to environmental changes and to recruit more diverse racial/ethnic populations. Lastly, future epigenetic wide association studies may be helpful in identifying other key regions that may impact epigenetic regulation of PUFA metabolism and biosynthesis in humans.

## Conclusions

In summary, we observed significant ASM between SNP rs174537 and 5 out of the 6 selected CpG sites across an important *FADS* regulatory region. The strongest ASM was detected between rs174537 and the methylation status of three CpG sites located in the *FADS2* promoter region, from leukocyte samples. This study replicates previous findings of strong ASM with rs174537 in other human tissues (including liver) and illustrates the importance of SNPs such as rs174537 and those in high LD with it on revealing the epigenetic landscape of key regulatory regions within the *FADS* genomic region in humans. Furthermore, we observed significant associations between circulating n-6 LC-PUFAs and DNA methylation quantitated from CD4+ cells and leukocytes. These data support the use of such peripheral and accessible samples for future nutrition and diet studies (e.g., nutrigenomics) to study the impact of dietary PUFA exposure on epigenetic regulation of PUFA biosynthesis and metabolism. Further work is needed to elucidate the specific mechanisms by which dietary PUFAs influence the *FADS* genomic landscape in humans and how they influence inflammation and disease.

## Additional files

Additional file 1: **Table S1.** List of primers and sequencing probes for DNA methylation quantification via pyrosequencing. (DOCX 14 kb)

Additional file 2: **Figure S1.** Genotype at SNP rs174537 is associated with circulating n-6 LC-PUFAs. Serum (cohort 1) and plasma (cohort 2) n-6 LC-PUFA levels are illustrated as mean ± SEM. (A) %DGLA, (B) %ARA, and (C) ARA/DGLA ratio. Asterisks represent statistically significant differences between genotypes (*p* < 0.05). (TIFF 175 kb)

Additional file 3: **Table S2.** Summary of plasma and serum fatty acids. Medians and IQR reported. Definitions of SFAs, MUFAs, PUFAs, and n-3/n-6 ratios are provided at the bottom of the table. (DOCX 18 kb)

## Abbreviations

ALA: α-Linolenic acid, 18:3n-3
ARA: Arachidonic acid, 20:4n-6
ASM: Allele-specific methylation
BH-FDR: Benjamini-Hochberg false discovery rate
CpG: Cytosine-(phosphate)-guanine
DGLA: Dihomo-γ-linolenic acid, 20:3n-6
DHA: Docosahexaenoic acid, 22:6n-3
DNA: Deoxyribonucleic acid
DPA: Docosapentaenoic acid, 22:5n-3
EPA: Eicosapentaenoic acid, 20:5n-3
eQTL: Expression quantitative trait loci
FADS: Fatty acid desaturase
FAME: Fatty acid methyl ester
GC-FID: Gas chromatography with flame ionization detection
GLA: γ-Linolenic acid, 18:3n-6
GWAS: Genome-wide, allele-specific
HDAC4: Histone acetylase 4
IQR: Interquartile range
LA: Linoleic acid, 18:2n-6c
LC-PUFAs: Long-chain polyunsaturated fatty acids
LD: Linkage disequilibrium
MUFA: monounsaturated fatty acid
n-3: Omega-3
n-6: Omega-6
NSAIDs: Nonsteroidal anti-inflammatory drugs
PBMC: Peripheral blood mononuclear cell
PCR: Polymerase chain reaction
PUFA: Polyunsaturated fatty acid
RNA: Ribonucleic acid
SFA: Saturated fatty acid
SNP: Single nucleotide polymorphism
