# Supplementary material for: Allele-specific methylation in the FADS genomic region in DNA from human saliva, CD4+ cells, and total leukocytes
Source: Clin Epigenetics. 2018 Apr 6;10:46. doi: 10.1186/s13148-018-0480-5 (PMC5889567; doi:10.1186/s13148-018-0480-5)
Supplement: Supplementary file 1 — Table S1. List of primers and sequencing probes for DNA methylation quantification via pyrosequencing. (DOCX 14 kb) [file 13148_2018_480_MOESM1_ESM.docx]

**Additional file 1: Table S1. List of primers and sequencing probes for DNA methylation quantification via pyrosequencing.**

|  | **CpG Site** | **Primers** |
| --- | --- | --- |
| *FADS1* Promoter | chr11:61584836 | PCR: 5’- AGAGGTAAATAGGGTTATAAAAATTGTG-3’; 3’-CCTCCAAAATTAAAAACTACTACCTACTTA-5’ |
|  | chr11:61584894 | Sequencing: AGTTTTTTTTTAATTAATTATTAAGT  (Custom order from Qiagen) |
| Putative Enhancer Region | chr11:61587979  (cg27386326) | PCR and Sequencing primer sets:  Commercially available from Qiagen (Cat# PM00609672) |
| *FADS2*  Promoter | chr11:61594865 | PCR: 5’-ATTGAGTTTATTGAGATTAGGGTAAGG-3’; 3’-ACTTTAAACCCTCTAATCAAACAATCTT-5’ |
|  | chr11:61594876 | Sequencing: CCTCTAATCAAACAATCTTAAAA  (Custom order from Qiagen) |
|  | chr11:61594907 |  |
