# Supplementary figures and images for: Allele-specific methylation in the FADS genomic region in DNA from human saliva, CD4+ cells, and total leukocytes
Source: Clin Epigenetics. 2018 Apr 6;10:46. doi: 10.1186/s13148-018-0480-5 (PMC5889567; doi:10.1186/s13148-018-0480-5)

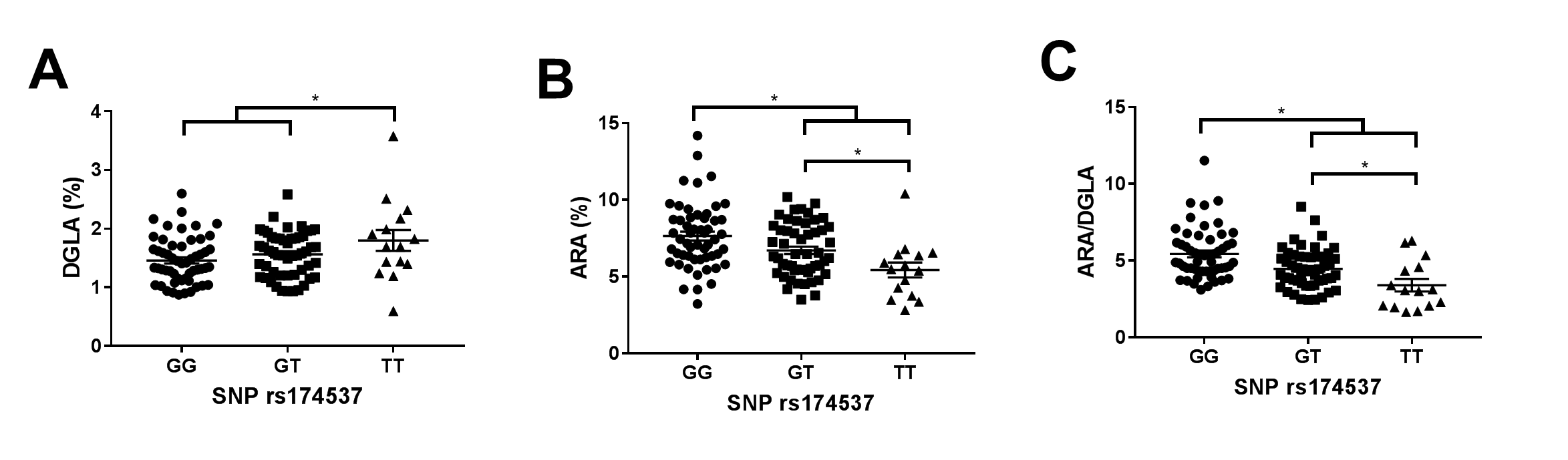

Supplement: Supplementary file 2 — Figure S1. Genotype at SNP rs174537 is associated with circulating n-6 LC-PUFAs. Serum (cohort 1) and plasma (cohort 2) n-6 LC-PUFA levels are illustrated as mean ± SEM. (A) %DGLA, (B) %ARA, and (C) ARA/DGLA ratio. Asterisks represent statistically significant differences between genotypes (p < 0.05). (TIFF 175 kb) [file 13148_2018_480_MOESM2_ESM.tif]
