# Supplementary material for: Allele-specific methylation in the FADS genomic region in DNA from human saliva, CD4+ cells, and total leukocytes
Source: Clin Epigenetics. 2018 Apr 6;10:46. doi: 10.1186/s13148-018-0480-5 (PMC5889567; doi:10.1186/s13148-018-0480-5)
Supplement: Supplementary file 3 — Table S2. Summary of plasma and serum fatty acids. Medians and IQR reported. Definitions of SFAs, MUFAs, PUFAs, and n-3/n-6 ratios are provided at the bottom of the table. (DOCX 18 kb) [file 13148_2018_480_MOESM3_ESM.docx]

| **Additional file 3: Table S2. Summary of plasma and serum fatty acids. Medians and IQR reported. Definitions of SFAs, MUFAs, PUFAs and n-3/n-6 ratios are provided at the bottom of the table.** | | | |
| --- | --- | --- | --- |
| **% Fatty Acid** | **Overall**    **(N=121)** | **Cohort 1**  **(Serum, fasting)**  **(N=89)** | **Cohort 2**  **(Plasma, fasting)**  **(N=32)** |
| ***Myristic, C14:0*** | 0.76 (0.64, 0.99) | 0.74 (0.58, 0.99) | 0.82 (0.67, 1.14) |
| ***Palmitic, C16:0*** | 21.45 (20.16, 23.09) | 21.90 (20.54, 23.48) | 20.71 (19.85, 21.85) |
| ***Palmitoleic, C16:1*** | 1.62 (1.26, 2.18) | 1.51 (1.21, 2.14) | 1.68 (1.42, 2.26) |
| ***Stearic, C18:0*** | 7.73 (7.11, 8.37) | 7.92 (7.41, 8.55) | 7.11 (6.64, 7.60) |
| ***Oleic, C18:1n-9c*** | 19.15 (17.42, 20.89) | 19.28 (17.72, 21.04) | 18.48 (17.24, 20.54) |
| ***LA, C18:2n-6*** | 33.95 (31.34, 36.85) | 34.26 (32.11, 37.07) | 32.47 (30.07, 35.89) |
| ***GLA, C18:3n-6*** | 0.38 (0.26, 0.57) | 0.33 (0.21, 0.45) | 0.61 (0.44, 0.76) |
| ***ALA, C18:3n-3*** | 0.57 (0.48, 0.70) | 0.55 (0.48, 0.66) | 0.64 (0.54, 0.84) |
| ***DGLA, C20:3n-6*** | 1.54 (1.21, 1.83) | 1.42 (1.17, 1.71) | 1.76 (1.49, 1.95) |
| ***ARA, C20:4n-6*** | 6.56 (5.52, 8.32) | 6.31 (5.28, 7.82) | 8.64 (7.15, 9.48) |
| ***EPA, C20:5n-3*** | 0.45 (0.35, 0.76) | 0.43 (0.33, 0.76) | 0.53 (0.38, 0.80) |
| ***Adrenic, C22:4n-6*** | 0.21 (0.15, 0.27) | 0.18 (0.13, 0.22) | 0.27 (0.23, 0.34) |
| ***DPA, C22:5n-3*** | 0.37 (0.28, 0.55) | 0.34 (0.25, 0.47) | 0.56 (0.48, 0.63) |
| ***DHA, C22:6n-3*** | 1.25 (0.97, 1.58) | 1.18 (0.81, 1.51) | 1.47 (1.19, 1.80) |
| ***Fatty acid ratios and sums*** | |  |  |
| *n-3/n-6* | 0.28 (0.24, 0.39) | 0.29 (0.24, 0.39) | 0.28 (0.24, 0.40) |
| *SFAs* | 30.09 (28.66, 32.71) | 30.68 (28.98, 33.03) | 28.83 (27.49, 29.87) |
| *MUFAs* | 21.12 (19.38, 23.09) | 21.16 (19.35, 23.09) | 20.89 (19.71, 23.28) |
| *PUFAs* | 46.87 (42.95, 49.04) | 46.51 (42.75, 48.24) | 48.20 (45.15, 49.61) |
| *n-3=EPA+DPA+DHA; n-6=ARA+Adrenic* | | | |
| *SFAs = myristic (14:0) + pentadylic (15:0) + palmitic (16:0) + stearic (18:0) + arachidic (20:0) + behenic (22:0) acids* | | | |
| *MUFAs = myristoleic (14:1) + palmitoleic (16:1) + heptadecenoic (17:1) + elaidic (18:1n9t) + oleic (18:1n9c) + euricic (22:1) acids* | | | |
| *PUFAs = LA + GLA + ALA + DGLA + ARA + EPA + Adrenic + DPA + DHA* | | | |
